# Ultrasound, Acetic Acid, and Peracetic Acid as Alternatives Sanitizers to Chlorine Compounds for Fresh-Cut Kale Decontamination

**DOI:** 10.3390/molecules27207019

**Published:** 2022-10-18

**Authors:** Maria Clara de Moraes Motta Machado, Bárbara Morandi Lepaus, Patrícia Campos Bernardes, Jackline Freitas Brilhante de São José

**Affiliations:** 1 Postgraduate Program in Nutrition and Health, Federal University of Espírito Santo, Marechal Campos Avenue, Vitoria 29047-105, ES, Brazil; 2 Department of Food Engineering, Center of Agrarian Sciences and Engineering, Federal University of Espírito Santo, Alegre 29500-000, ES, Brazil; 3 Department of Integrated Health Education, Federal University of Espírito Santo, Marechal Campos Avenue, Vitoria 29047-105, ES, Brazil

**Keywords:** ultrasound, acetic acid, peracetic acid, disinfection, vegetables, ready-to-eat, food safety, non-chlorine sanitizer, emerging technologies

## Abstract

Chlorinated compounds are usually applied in vegetable sanitization, but there are concerns about their application. Thus, this study aimed to evaluate ultrasound (50 kHz), acetic acid (1000; 2000 mg/L), and peracetic acid (20 mg/L) and their combination as alternative treatments to 200 mg/L sodium dichloroisocyanurate. The overall microbial, physicochemical, and nutritional quality of kale stored at 7 °C were assessed. The impact on *Salmonella enterica* Typhimurium was verified by plate-counting and scanning electron microscopy. Ultrasound combined with peracetic acid exhibited higher reductions in aerobic mesophiles, molds and yeasts, and coliforms at 35 °C (2.6; 2.4; 2.6 log CFU/g, respectively). Microbial counts remained stable during storage. The highest reduction in *Salmonella* occurred with the combination of ultrasound and acetic acid at 1000 mg/L and acetic acid at 2000 mg/L (2.8; 3.8 log CFU/g, respectively). No synergistic effect was observed with the combination of treatments. The cellular morphology of the pathogen altered after combinations of ultrasound and acetic acid at 2000 mg/L and peracetic acid. No changes in titratable total acidity, mass loss, vitamin C, or total phenolic compounds occurred. Alternative treatments presented equal to or greater efficacies than chlorinated compounds, so they could potentially be used for the decontamination of kale.

## 1. Introduction

Fresh-cut or minimally processed vegetables are characterized as small portions that are ready to eat (RTE) or to cook [1]. Consuming fresh leafy vegetables, such as lettuce, spinach, cabbage, and kale, has certain benefits for human health [2,3], and the interest in healthy and well-balanced diets has increased. This interest includes a higher demand for foods such as RTE vegetables [1,4]. Kale is a leafy vegetable in the *Brassica* group that contains many important nutrients, such as calcium, iron, and vitamins A, C, K, and B5, and can be commercialized as an RTE vegetable. Vegetables are usually consumed as an excellent source of phenolic compounds that are known for their antioxidant activity [5].

The main limitations of RTE products are related to the fact that they are much more expensive than their conventional counterparts. In contrast, as an advantage, they often do not require an additional preparation step before consumption. However, the lack of a formal cooking or preparation step can cause concerns for consumers regarding the microbiological, sensorial, and nutritional qualities of the produce [6,7]. Additionally, the cases of foodborne disease outbreaks associated with the consumption of RTE leafy vegetables contaminated with pathogens like *Escherichia coli*, *Salmonella* spp., and *Listeria* spp. have increased over the years [8].

For that reason, cleaning and sanitization are crucial steps to guaranteeing the microbiological safety of RTE vegetables. Several compounds have been widely studied as alternatives to chlorine-based cleaning products, which are the most used chemicals for the sanitization of vegetables by the food industry [9]. Among the newly explored compounds are organic acids, such as acetic acid (AA) and lactic acid (LA), and peracetic acid (PAA), which have advantages over-chlorinated compounds when used as antimicrobial agents [10,11,12,13,14,15,16,17,18]. Because sanitization with organic molecules does not generate toxic or carcinogenic residues, AA and PAA can be used as alternative chlorinated compounds in the ready-to-eat produce industry [11,16].

Some technologies, such as ultrasound (US), can be combined with chemical cleaners or by themselves for fruit and vegetable decontamination post-harvest [3,12,17]. US is described as sound waves above the human hearing limit that could be applied in several industrial functions, such as food processing [19,20]. US is classified as a physical and nonthermal technology that can prolong shelf life and reduce the microbial counts of heat-sensitive foods to preserve the nutritional and sensorial characteristics of products [1,2,4]. The mechanisms of inactivation of microorganisms by US have been attributed to cavitation, reducing bacterial viability by weakening cell walls and facilitating access to sanitizers [3,17].

Considering the hurdle theory, we hypothesize that US combined with organic acids can generate a synergistic reduction in microorganisms and still preserve food quality. Some studies have investigated the effect of sanitization with organic acids and US, isolated or combined, on the microbiological and physicochemical quality of vegetables [10,11,13,18]. However, studies about the possible synergistic effects of US and organic acids compared to chlorine compounds on the overall quality of RTE kale are still scarce.

Therefore, the present study investigated the microbiological and physicochemical quality of fresh-cut kale subjected to sodium dichloroisocyanurate and alternative sanitization procedures. We aimed to verify four hypotheses in this study: (i) AA and PAA would show similar or better effectiveness in the disinfection of fresh-cut kale than the chlorine-based sanitizer; (ii) the combination of US with AA and PAA would improve the chemical sanitizer’s efficiency; (iii) the treatments would contribute to the preservation of physicochemical properties, and; (iv) the treatments proposed in this study would not have a detrimental effect on bioactive compounds.

## 2. Material and Methods

### 2.1. Experimental Design, Obtaining Samples, and Sanitizing Procedure

The experiment was conducted in a completely randomized design, and three experimental repetitions, with duplicates, were conducted for each sanitizing treatment. The treatment conditions chosen were based on previous studies and literature.

**Figure 1:**
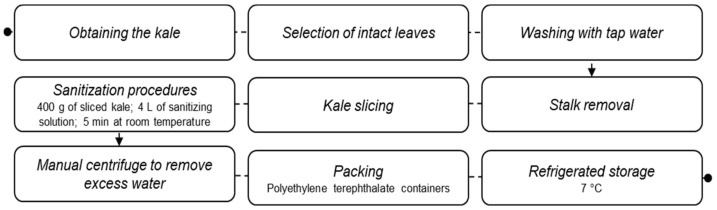
Flowchart of obtaining and processing minimally processed (ready-to-eat) kale used in this study.

Kale (Brassica oleracea var. acephala) was obtained from a local market, stored at 7 °C for a maximum of 24 h, and processed according to Figure 1. Any leaves that were damaged and/or yellowish were discarded, and the rest were washed in tap water to remove soil adhered to the leaf surface. The sanitization step consisted of the immersion of 400 g of sliced kale in 4 L of sanitizing solution for 5 min at room temperature. The effects of 1000 mg/L and 2000 mg/L acetic acid (Neon^®^, São Paulo, Brazil), 20 mg/L peracetic acid (Nippon Chemical^®^, São Paulo, Brazil), and ultrasound (bath-type, 50 kHz, 150 W, model NI 1207, Nova Instruments^®^, São Paulo, Brazil) were evaluated. Moreover, the combination of organic acids sanitizers with ultrasound was tested. The reference treatment was a 200 mg/L sodium dichloroisocyanurate solution (Nippon Chemical^®^, São Paulo, Brazil). The control vegetable sample was only washed in tap water and not subjected to any sanitization procedure.

### 2.2. Storage Time Study

After the sanitization procedures were carried out, the kale samples were packed in polyethylene terephthalate containers and stored at 7 ± 1 °C. The assessment of sanitizers, ultrasound, and their combination effects on natural microbiota and physicochemical quality were evaluated immediately after sanitization and after 6 days of storage at 7 ± 1 °C, as described in the following paragraphs. Salmonella populations intentionally inoculated on kale surfaces were analyzed only after sanitizing treatments (first day).

### 2.3. Evaluation of the Efficiency of Sanitization in the Natural Microbiota

This step was performed according to the methodology of the American Public Health Association (APHA) [21]. Kale samples (25 g), previously subjected to sanitizer treatments but not inoculated with any pathogen, were homogenized with 225 mL of 0.1 % peptone water in a stomacher (Marconi^®^, Piracicaba, São Paulo, Brazil) for 2 min at normal speed. Appropriate decimal dilutions were prepared, and aliquots were transferred to specific culture media for the determination of the counts of each microbial group. The plating of the aliquots was performed in duplicates, and the results were expressed in colony-forming units per gram (CFU/g). Mesophilic aerobic bacteria were plated on plate count agar (Himedia^®^, Mumbai, India) and incubated at 35 °C for 48 h. Molds and yeasts were plated on potato dextrose agar (Himedia^®^, Mumbai, India) and incubated at 25 °C for 7 days. Coliform counts were carried out at 35 °C using Petrifilm plates (3M^®^, Sant Paul, MN, USA), incubated at 35 °C for 48 h, enumerating *E. coli* as blue colonies with gas and total coliforms at 35 °C as red colonies with gas.

### 2.4. Inoculation of Salmonella enterica Typhimurium ATCC 14028 on Kale Surface

This experimental step was carried out separately from the natural contamination evaluation and was performed according to previous studies [11,22], with some modifications. Suspensions of vegetative Salmonella enterica Typhimurium ATCC 14028 cells were activated two consecutive times in 10 mL of brain heart infusion broth (BHI; Himedia^®^, Mumbai, India) and incubated at 37 °C for 24 h until populations of 10^8^ and 10^9^ CFU/mL were reached. Kale leaves (25 g) were cut into 3 cm × 3 cm pieces and placed in previously sterilized plastic bags. Then, the inoculum (BHI inoculated with vegetative cells) was added along with 225 mL of 0.1 % peptone water, and the plastic bags containing the inoculum and the vegetable material were stirred gently for 5 min. The content was kept in static contact with the cell suspension for 60 min at 24 ± 1 °C to promote higher cell adhesion to the vegetable. After, the cell suspension was drained, and the kale contaminated with the pathogen was subjected to the same sanitization treatments described before. After each treatment, the samples were transferred to sterile plastic bags containing 0.1% peptone water and then homogenized in a stomacher (Marconi^®^, Piracicaba, São Paulo, Brazil) for 2 min at normal speed. Finally, 1 mL of homogenized sample was used to prepare decimal dilutions, and aliquots were plated on Hektoen agar (Himedia^®^, Mumbai, India). After plating and incubation at 37 °C for 24 h, colony counting was performed.

### 2.5. Scanning Electronic Microscopy after Inoculation of Salmonella enterica Typhimurium ATCC 14028 and Decontamination Procedures

Scanning electron microscopy was performed to confirm the adhesion of Salmonella cells and the possible effect of their sanitization on the kale surface. First, the preparation of samples for microscopic observation was conducted. Briefly, kale inoculated with Salmonella Typhimurium ATCC 14028 strain and sanitized with different procedures were cut into 1.0 cm × 1.0 cm pieces using a previously sterilized scalpel. Kale slices were rinsed in 0.1 mol/L phosphate-buffered saline (PBS) at pH 6.8 to 7.2 to remove residues from sanitizers and unbound cells. The fixation step consisted of treatment with 5% glutaraldehyde (Vetec^®^, São Paulo, Brazil) and 0.1 mol/L PBS buffer in a 1:1 ratio for 1 h at room temperature. Thereafter, the samples were washed 6 times in 0.1 mol/L PBS buffer at pH 6.8 to 7.2, and each wash lasted for 10 min. The dehydration stage consisted of serial treatments in 30, 50, 70, 80, and 95 °C Gay-Lussac (GL) ethanol for 10 min each and three 15 min treatments in 100 °C GL ethanol. Samples were transferred to a critical point dryer model AutoSamdri-815 A (Tousimins^®^, Rockville, MD, USA) to complete the dehydration process. Finally, they were submitted to a Desk V sputter coater (Denton Vacuum^®^, Cherry Hill, NJ, USA) for deposition of a thin layer of gold and then analyzed by scanning electron microscopy (model JSM-6610, Scanning Electron Microscope^®^ LV, JEOL, Tokyo, Japan).

### 2.6. Physicochemical and Bioactive Compound Analysis

The total titratable acidity, pH, total soluble solids, and vitamin C content were determined according to the AOAC [23]. The total phenolic compounds were measured using the Folin–Ciocalteu reagent (Sigma Aldrich^®^, St. Louis, MO, USA) [24]. The effect of sanitizing treatments on antioxidant capacity was evaluated using 1,1-diphenyl-2-picrylhydrazyl (DPPH) (Sigma Aldrich^®^, St. Louis, MI, USA), according to Blois [25]. The percentage of mass lost by each sample was determined according to Equation (1):

(1) $$\%\;Mass\;loss\;\left(t\right)=\frac{\left[M_{0}-M_{t}\right]\times 100}{M_{0}}\;$$

where % *Mass loss* (*t*) is the mass lost after time *t*; *M*_0_ is the initial mass of the sample; and *M_t_* is the weight of the sample at time *t*.

These analyses identified possible changes in the quality of the vegetables after sanitization treatments compared to non-sanitized samples (control). The samples used in this series of tests were not inoculated with the pathogen.

### 2.7. Statistical Analysis

The efficiency of the sanitization treatments in changing the microbiological and physicochemical characteristics was evaluated soon after sanitization and at the end of the storage period using the paired *t*-test. For the evaluation of the treatments at the time point, the data were analyzed by analysis of variance (ANOVA), and the means were compared by Tukey’s test at 5% probability. All results were analyzed with the InfoStat Statistical Software for students (version 2012, Cordoba National University, Cordoba, Argentina).

## 3. Results and Discussion

### 3.1. Effect of Sanitization Treatments on the Natural Contaminating Microbiota

The results of the entire discussion in this section are shown in Table 1. None of the samples was naturally contaminated with *E. coli*. All applied treatments, except US 50 kHz, significantly reduced the count of aerobic mesophiles (*p* ≤ 0.05). The sanitization treatments that promoted the highest reductions in aerobic mesophile counts at day 1 compared to sodium dichloroisocyanurate were US combined with 2000 mg/L AA (2.5 log CFU/g reduction) and US combined with 20 mg/L PAA (2.6 log CFU/g reduction) (*p* ≤ 0.05).

US alone did not promote a statistically significant reduction in aerobic mesophiles compared to non-sanitized kale samples (*p* > 0.05). Other studies have also shown that US alone is less effective in microbial inactivation, but when applying US combined with chemicals, the bactericidal effect increases [2,10,12,26]. Duarte et al. [2] verified that US alone and US combined with benzalkonium chloride promoted reduction equal to 0.6 and 2.5 log CFU/g of mesophilic aerobic bacteria counts on purple cabbage, respectively. Alvarenga et al. [26] verified that the application of ultrasound alone reduced the aerobic mesophile count by 1.09 log CFU/g in strawberries. The limited efficiency of US treatment isolated in the sanitization step of fruits and vegetables could be attributed to the capacity of microorganisms to penetrate these foods, becoming inaccessible to sound waves and the cavitation process produced by the equipment [7]. Furthermore, treatment conditions, such as time, ultrasound equipment (frequency and potency), and the type and amount of food treated, could impact sanitization efficiency [2].

Increasing the concentration of AA had a small impact on the reduction in aerobic mesophilic bacteria, molds, yeasts, and coliforms at 35 °C. The results demonstrate that it is still possible to produce microbiologically safe vegetables minimizing the concentration of chemical sanitizers. Furthermore, the use of a lower concentration may be more viable from an economic point of view. Similar results were described previously [11] after the sanitization of arugula leaves with AA for aerobic mesophile counts. However, the authors described an improvement in the reduction in molds and yeasts after the increase in organic acid concentration.

In the present study, the treatments in which the organic acids were applied in isolation or combined with US did not differ statistically (*p* > 0.05), indicating that there was no incremental reduction in the counts of microorganisms. In this way, the combined treatments had similar effects to the chemical treatments alone. Therefore, no synergistic effect was observed on the first day when ultrasound was combined with AA and PAA for all microorganism groups evaluated. However, treatments combining US with organic acids produced samples with stable aerobic mesophile counts over the storage period, different from what was observed for other treatments. These results indicate that treatments with US and organic acids are able to prevent microbial multiplication during the storage period.

Concerning molds and yeasts, all the treatments applied provided significant reductions (*p* ≤ 0.05), and further reduction beyond what is achieved by sodium dichloroisocyanurate (*p* ≤ 0.05) was observed when US was applied in combination with both concentrations of AA and PAA. On the sixth day of storage, kale samples that had been submitted to the different sanitization treatments had a statistically significant number of molds and yeasts.

Some postharvest practices such as peeling and slicing can damage the vegetal tissue, favors the release of cellular fluids, and provides water and nutrients to microorganisms’ growth [7]. Despite this fact, the counts on the sixth day of storage were statistically the same as the counts determined one day after sanitization (*p* > 0.05). This result indicates that there was no resumption of growth of this group of microorganisms during storage.

Cao et al. [27] achieved a reduction in molds and yeasts after the application of US at 40 kHz for 10 min in strawberries. In the present study, five minutes of sanitization was sufficient to reduce the counts of molds and yeasts of kale, and this result may be associated with the use of US at a higher frequency (50 kHz). However, it is essential to note that the effects of ultrasound depend, in addition to the treatment conditions applied (e.g., temperature, the amplitude of the wave, time, and frequency), on the type, volume, and composition of food being sanitized; its properties (e.g., surface roughness); and the interactions between the surface and microorganisms [9,22].

For coliforms at 35 °C, US with PAA afforded a statistically superior reduction (*p* ≤ 0.05) compared to treatment with sodium dichloroisocyanurate, 2000 mg/L AA, and US alone (Table 1) on the first day of analysis. The reduction after the application of US combined with PAA was 2.6 log CFU/g. On the other hand, the US and SDC 200 mg/L promoted the lowest reductions, equal to 0.8 and 1.2 log CFU/g, respectively. On day 6, no coliform at 35 °C was identified in the sanitized samples, even in the smallest plated dilution (10^−1^). This result may suggest that the sanitization treatments caused irreparable damage to the cells, and they could not remain throughout the storage time; that is, the treatments were all effective in the inactivation of coliforms at 35 °C.

Leafy vegetable composition favors the presence and growth of many pathogenic and non-pathogenic microorganisms, including bacteria, yeasts, and molds. Considering the long distance between farms and commercialization, the procedures applied during processing must guarantee the safety of the product [7].

In the present work, the reductions obtained for the natural microbial groups after sanitization with organic acids were similar to or higher than those observed with conventional chlorinated compound treatment.

Chlorinated compounds are oxidative disinfectants, and in contact with water, these compounds mainly produce hypochlorous acid, but other reactive chlorine species as well. These subproducts damage multiple cellular components of the cell, and this is the mechanism of action [7]. However, chlorinated compounds generally have limited antimicrobial action, and under typical conditions, their reduction values reach approximately 2 log CFU/g. Chlorinated compounds are widely used by the food industry, mainly because they are inexpensive, but there are concerns related to the interaction with the organic components of food and the formation of toxic and potentially carcinogenic trihalomethanes, which have the potential to cause skin irritation [9,28].

In contrast, PAA is an oxidative disinfectant, environmentally friendly, and effective in inactivating aerobic and pathogenic bacteria and molds as well as yeasts at concentrations even lower than those required for chlorinated solutions, without a report on cytotoxic effects [7]. Moreover, PAA cannot be deactivated by enzymes such as catalase and peroxidase [29], and it decomposes into nontoxic residues (AA and oxygen) [9]. Furthermore, its concentration is comparatively stable in the presence of high organic loads because of its slow reaction degree with organic matter in the wash water [16]. The antimicrobial effect of PAA is related to the release of reactive oxygen species and DNA and lipid damage [9].

Organic acids are naturally occurring and generally recognized as safe (GRAS) compounds. The mechanism of microbial inactivation is attributed to their pKa values since these compounds are considered weak organic acids, and their bactericidal effect is higher than strong organic acids. It occurs because they are more lipophilic, and it facilitates the penetration in the bacterial cells, causing faster acidification of the interior, reducing the pH, and inhibiting essential metabolic reactions [9].

### 3.2. Inactivation of Salmonella enterica Typhimurium and Microscopic Visualization of Cells Adhered to the Surface of Kale after Sanitization

The count of S. enterica Typhimurium cells on the non-sanitized kale samples was 6.3 ± 0.1 log CFU/g, indicating that this pathogenic bacterium can adhere to the surface of kale. A point that needs to be addressed is that biofilm formation starts with microbial adhesion to the plant. Bacteria that have flagella, for example, may show higher swimming motility for surface colonization after adhesion. Salmonella spp. is a group of bacteria capable of forming biofilms, and the presence of fimbriae in this group is crucial to intensifying the degree of virulence of the pathogen after biofilm formation [7,30]. Furthermore, pathogens produce some compounds such as polysaccharides (e.g., cellulose), lipopolysaccharides, and colonic acid to colonize plant surfaces [7].

**Figure 2:**
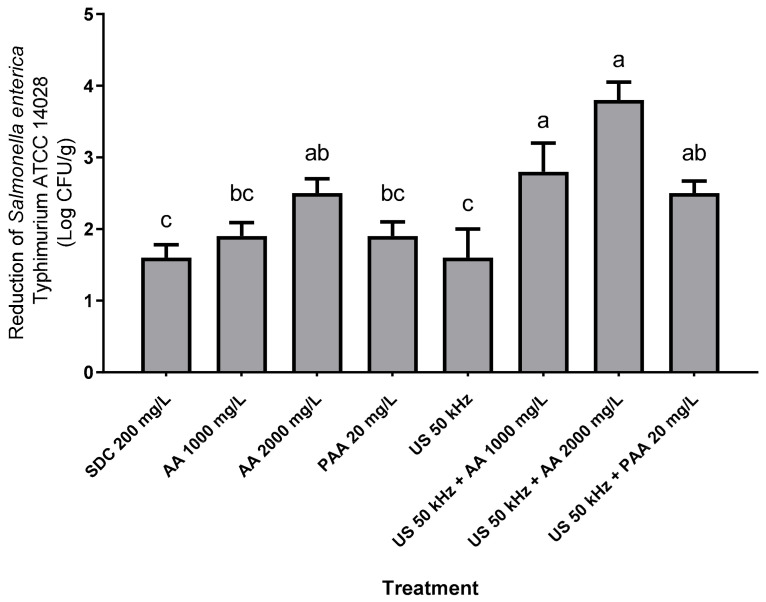
Decimal reductions in the count of *Salmonella enterica* Typhimurium ATCC 14028 (log CFU/g) adhered to the surface of kale compared with the non-sanitized sample. Treatments (columns) with the same letter do not differ significantly by Tukey’s test after three replications (*p* > 0.05). SDC: sodium dichloroisocyanurate; AA: acetic acid; PAA: peracetic acid; US: ultrasound.

After the sanitization of kale, all treatments reduced the counts by significantly more than 1.0 log CFU/g, and the average reduction in the pathogen in proposed treatments varied between 1.6 and 3.8 log CFU/g (Figure 2). Furthermore, all alternative treatments applied in contaminated samples reduced the pathogen equal to or higher than sodium dichloroisocyanurate. In the present study, the application of 50 kHz US without any chemical treatment caused a reduction that was statistically equal to treatments with sodium dichloroisocyanurate, PAA, and AA without the US (*p* > 0.05). Different results were observed by Lepaus et al. [11], who evaluated the efficacy of 200 mg/L sodium dichloroisocyanurate and 200 mg/L sodium hypochlorite in the sanitization of strawberries, cucumbers, and arugula leaves. The results demonstrated that chlorinated compounds were less effective than organic acids and hydrogen peroxide in reducing natural microbiota and Salmonella Enteritidis. São José and Vanetti [31] also observed that 200 mg/L sodium dichloroisocyanurate did not inactivate Salmonella enterica Typhimurium inoculated in cherry tomatoes. Alenyorege et al. [1] evaluated different conditions for fresh-cut Chinese cabbage decontamination and observed that US (40 kHz, 125.45 W/L for 15 min) promoted reduction equal to 5.6 and 4.7 log CFU/g for *E. coli* and *L. innocua*, respectively.

In the present study, the treatments using US combined with AA at 2000 mg/L, US combined with AA at 1000 mg/L, and US associated with PAA 20 mg/L afforded reductions of 3.8, 2.8 log, and 2.5 CFU/g, respectively. Similar results are presented by Rosário et al. [32], who observed that the US + peracetic acid occasioned a reduction of 2.1 log of CFU/g of Salmonella enterica subsp. enterica on strawberries.

However, in the present study, no synergic effect was observed in the combination of US and organic acids on the Salmonella enterica Typhimurium inactivation. In contrast, Turhan et al. [33] observed that organic acid treatment combined with US produced mainly synergistic effects, and the highest value on the surface of lettuce was achieved when US and citric acid for 30 min were applied. According to Mendoza et al. [7], using a single technique is not sufficient for biofilm removal. Turhan et al. [33] mentioned that the combination of ultrasound with other processes produces synergistic and antagonistic impacts against pathogens. Ultraviolet light treatment (30 mW/cm^2^) combined with 80 mg/L of PAA, a recommended commercial practice for cleaning fresh produce, achieved significantly higher Salmonella spp. reduction in lettuce [16]. Rosário et al. [32] also observed that adhered Salmonella enterica could efficiently be removed from the surface of strawberries through the application of 40 mg/L PAA combined with 40 kHz US for 5 min. The intense pressure generated during US treatment can contribute to the penetration of chemical oxidants through the cell membrane, and the cavitation process can aid in the breakdown of microorganisms, which culminates in higher efficiency of the sanitization treatment [34].

**Figure 3:**
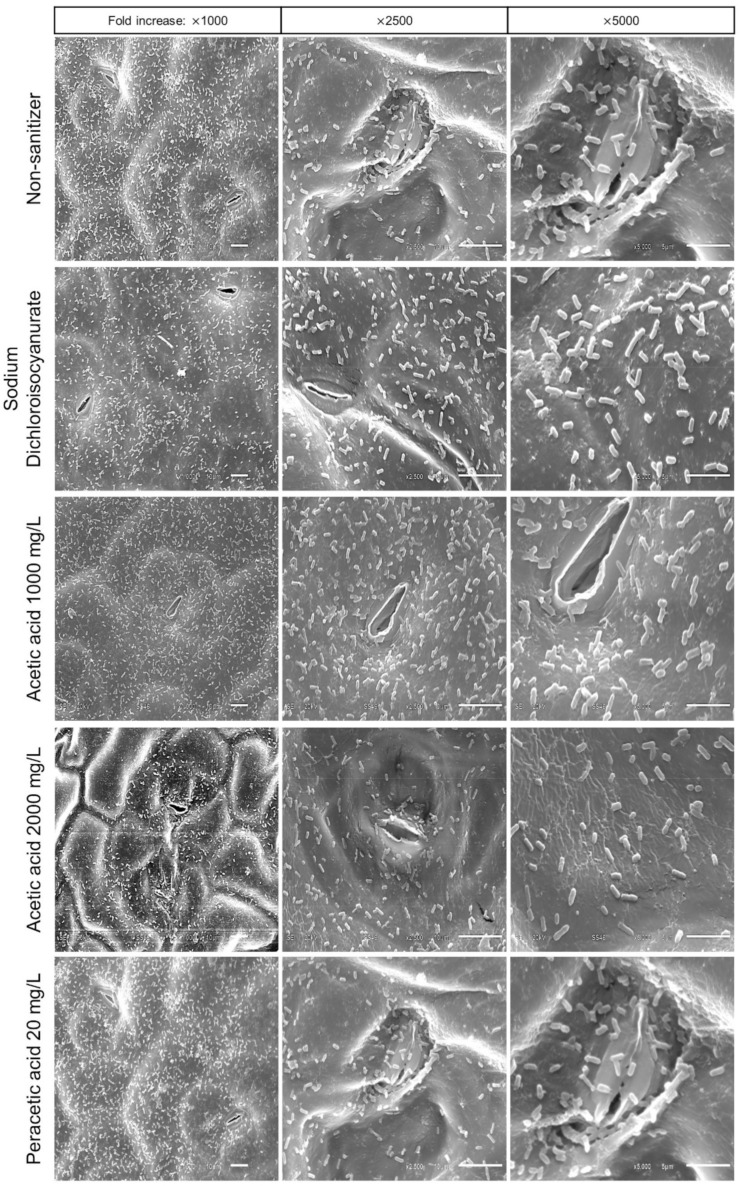
Scanning electronic microscopy micrographs of kale samples intentionally contaminated with *S.* Typhimurium cells ATCC 14028 and submitted to different sanitization treatments (Non sanitizer, sodium dichloroisocyanurate; Acetic Acid 1000 mg/L; Acetic Acid 2000 mg/L, Peracetic Acid 20 mg/L) for 5 min.

**Figure 4:**
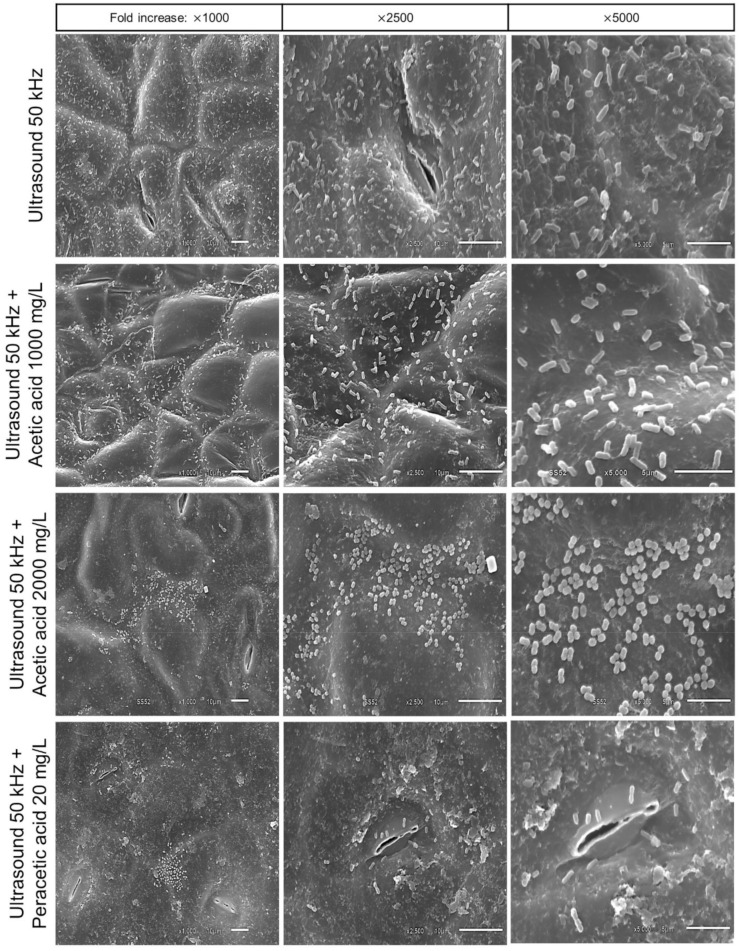
Scanning electronic microscopy micrographs of kale samples intentionally contaminated with *S.* Typhimurium cells ATCC 14028 and submitted to different sanitization treatments (Ultrasound 50 kHz, Ultrasound 50 kHz + Acetic Acid 1000 mg/L; Ultrasound 50 kHz + Acetic Acid 2000 mg/L; Ultrasound 50 kHz+ Peracetic Acid 20 mg/L) for 5 min.

Microscopy was applied to confirm microbial adhesion and identify the morphological appearance of the pathogen after the sanitization procedures. The adhesion of the Salmonella cells and the effect of proposed treatments on the surface of kale were confirmed by scanning electron microscopy (Figure 3 and Figure 4).

In Figure 3, some characteristics were observed in the bacterial adhesion after sanitization procedures and support the result described before for the Salmonella inactivation study. Visually, treatments with sodium dichloroisocyanurate, AA 1000 mg/L, and 50 kHz US appeared to have little impact on pathogen inactivation (Figure 3). Therefore, these conditions could not efficiently remove the Salmonella cells adhered to the kale surface. The survival of pathogenic microorganisms after sanitization treatments becomes a risk to food safety. However, when the combination of US with AA 2000 mg/L and PAA was applied, it was observed that these treatments reduced adherent Salmonella cells at the same time that the cellular morphology of the pathogen was altered (Figure 4). These results demonstrate the importance of properly evaluating and choosing the method that will be used.

Li et al. [35], when applying US (55 °C; 3–15 min) in Staphylococcus aureus cells, observed a great impact on the cell morphology, as well as leakage of cellular content, cell wall disintegration, and plasma membranes. US promotes microbial inactivation, generates membrane damage, disrupts the cell wall, and damages DNA [36]. Furthermore, the collapse of cavitation bubbles can cause pore formation and partial disruption of cell walls and cytoplasmic membranes [35].

Sanitizers that do not promote adequate inactivation of microorganisms on the surface of vegetables allow the possibility of microbial growth during storage. Despite the reduction in the number of natural microbiota cells, in the present study, the inability of some sanitizers to effectively remove the pathogen adhered to the vegetable surface promotes the possibility of cell survival and proliferation during storage. For future research, it is recommended that an analysis is also carried out during storage.

The ability of pathogens to adhere to leaf epidermises and form biofilms are challenges for many sanitization procedures and is thus detrimental to product safety. In addition, intrinsic food parameters (e.g., roughness and hydrophobicity), microorganisms (e.g., presence of fimbriae, flagella, and pili) [37], and the interactions between them (e.g., the free energy of hydrophobic interaction and free energy of adhesion of surfaces) can influence the decontamination efficiency [22]. According to Pimentel-Filho et al. [38], surfaces are susceptible to biofilm formation, and the surface physiochemical aspects present an important function in bacterial adhesion. Hydrophobic interactions between cell surface and substrate facilitate cells to surmount electrostatic repulsive forces and favor adhesion [39]. For this reason, it is essential to study processes that are capable of eliminating microorganisms and do not bring risks to consumer health and the environment.

### 3.3. Impact of Sanitization Treatments on the Physicochemical and Nutritional Parameters

Decontamination treatments in fresh produce could affect food quality, and methods should preserve characteristics at appropriate levels [33]. Regarding the physicochemical parameters (Table 2), both concentrations of treatments with AA, alone or associated with ultrasound, significantly reduced (*p* ≤ 0.05) the pH of the samples concerning non-sanitized kale. However, the other treatments presented results statistically similar to the chlorinated compound for this variable (*p* > 0.05). No statistical difference in pH values between samples occurred between the first and sixth day of storage, except for the kale treated with 2000 mg/L of AA. Furthermore, this treatment, isolated and combined with ultrasound, significantly increased (*p* ≤ 0.05) the titratable total acidity (TTA) values compared to the non-sanitized kale sample on the first day of storage. Through to the sixth day, no variations in TTA occurred.

São José et al. [40] observed a decrease in pH values of watercress and parsley treated with sodium dichloroisocyanurate (200 mg/L), hydrogen peroxide (5%), and PAA (40 mg/L) after 10 min of treatment. However, the pH of strawberries decreased only after sanitization with PAA. Similarly, another study demonstrated that strawberry and cucumber samples were more resistant to the 5 min immersion sanitization method with AA (1 and 2%), lactic acid (1 and 2%), and hydrogen peroxide (3%) than arugula leaves [18].

The changes in these parameters may indicate that the time of the procedure or the immersion sanitization method for leafy vegetables may have contributed to the incorporation of the sanitizing solution, reducing the pH value of the sample. Therefore, monitoring the pH values after sanitization, as well as the conditions applied during the procedure (e.g., time, temperature, concentration), is very relevant because it avoids the fast deterioration of the product during storage due to changes in the pH values after processing.

A statistically significant decrease (*p* ≤ 0.05) in the total soluble solids content occurred after the application of both concentrations of AA, as well as the treatments combining US with the organic acids. There was no variation in this parameter over the six days of refrigerated storage.

In the present study, the mass loss over refrigerated storage was not significantly affected by the applied treatments (*p* > 0.05), and the mean of samples was 1.56 ± 0.42 (data not shown). This result indicates that these values were not enough to cause a significant decline in product quality. Excessive water loss due to the transpiration of the vegetal tissue can contribute to the loss of mass and lead to nutritional losses, wilting, and changes in texture and aroma. According to Turhan et al. [33], US treatment can affect the firmness of fruits and vegetables, and different results could be associated with the processing conditions, food matrix, variety, maturity stage, intensity, and time of US.

No reduction in vitamin C content was observed in sanitized kale samples compared to non-sanitized vegetables (*p* > 0.05) on the first (mean 51.8 ± 5.0 mg of ascorbic acid/100 g) and last day (mean 47.9 ± 7.4 mg of ascorbic acid/100 g) of storage (Table 3).

Different from this result, Wu et al. [41] observed that the contents of ascorbic acid significantly decreased during 12 days of storage of bok choy treated with aqueous chlorine dioxide in combination with US treatment. Vitamin C is considered the least stable of all vitamins and can easily be destroyed during processing and storage, so it is a natural indicator of the quality of food-processing techniques. Despite this fact, in the present study, the vitamin C content was maintained in all the evaluated sanitization treatments. This maintenance in ready-to-eat products is necessary since consumers are aware that fresh and vitamin-rich products have health benefits.

The sanitization treatments and storage time did not cause significant changes in the level of total phenolic compounds in kale samples (*p* > 0.05) (Table 3). After sanitization procedures, the mean value for this nutritional parameter was 21.5 ± 2.3 mg gallic acid equivalent/100 g fresh sample. At the end of storage, the value was 23.9 ± 4.5 mg gallic acid equivalent/100 g fresh sample. This result is considered positive because even the application of chemical sanitizers in combination with US or on their own guaranteed that the content of these bioactive compounds in kale was maintained.

The antioxidant activity of kale was preserved after sanitization without differences between treatments (mean 90.8 ± 6.2%; *p* > 0.05), but during storage, it fell significantly (*p* ≤ 0.05) in all treatments (mean 39.2 ± 6.8%) (Table 3). The most important goal of sanitization is to reduce the number of pathogenic and spoilage microorganisms, but it is crucial to maintain the physicochemical and nutritional properties of the food.

If the operating conditions are inadequate, the nutritional quality of vegetables may be affected. Thus, it is important to adjust the time and sanitizer concentrations to obtain a balance that results in decontamination. Therefore, the procedures applied in this study and the packaging used were adequate to maintain the mass of the samples throughout the storage period, as well as the vitamin C and bioactive compounds. Storage in a temperature and humidity-controlled environment is recommended.

## 4. Conclusions

Considering the hypotheses tested in this study, we concluded that AA and PAA are good alternatives to chlorine-based compounds for kale sanitization, as they obtained a sanitizing effect similar or superior to these compounds in microbial reduction. However, no synergistic effect occurred after the combination of US and AA and PAA for all microorganisms. Moreover, despite the reduction in pH by treatments with alternative strategies, the treatments contributed to the preservation of the physicochemical and nutritional properties of kale. Finally, we concluded that the proposed treatments have the potential to be applied in the sanitization step of kale, but other methods, such as spraying techniques, are recommended over immersion for this crop. Studies that evaluate different conditions of treatment with ultrasound, concentrations of organic acids, and sanitization time are suggested. Furthermore, further studies should be conducted to evaluate the sensory quality of sanitized vegetables to verify if characteristics are retained at satisfactory levels after the application of decontamination strategies.

## Figures and Tables

**Table 1:** Average and standard deviation of aerobic mesophilic bacteria, molds, yeasts, and coliforms at 35 °C counts (log CFU/g) in kale samples after sanitization and over storage at 7 °C.

Treatment	Aerobic Mesophilic Bacteria		Molds and Yeasts		Coliforms at 35 °C	
	Day 1	Day 6	Day 1	Day 6	Day 1	Day 6
Non-sanitizer	6.8 ± 0.3 ^aA^	8.4 ± 0.1 ^bA^	5.4 ± 0.3 ^aA^	5.5 ± 0.5 ^aA^	3.8 ± 0.3 ^aA^	2.00 ± 0.19 ^b^
SDC 200 mg/L	5.4 ± 0.4 ^aBC^	8.1 ± 0.3 ^bA^	4.2 ± 0.3 ^aBC^	4.5 ± 0.5 ^aAB^	2.6 ± 0.2 ^B^	Nd
AA 1000 mg/L	5.0 ± 0.5 ^aBCD^	7.7 ± 0.3 ^bA^	3.6 ± 0.3 ^aBCD^	4.0 ± 0.2 ^aAB^	2.2 ± 0.3 ^BC^	Nd
AA 2000 mg/L	4.5 ± 0.5 ^aCD^	5.8 ± 0.8 ^bAB^	3.5 ± 0.2 ^aCD^	3.9 ± 0.3 ^aAB^	2.3 ± 0.3 ^B^	Nd
PAA 20 mg/L	5.0 ± 0.3 ^aBCD^	6.7 ± 0.9 ^bAB^	3.6 ± 0.3 ^aBCD^	3.7 ± 0.3 ^aB^	2.0 ± 0.5 ^BC^	Nd
US 50 kHz	5.7 ± 0.4 ^aAB^	7.4 ± 0.6 ^bAB^	4.4 ± 0.3 ^aB^	4.5 ± 0.6 ^aAB^	2.9 ± 0.6 ^AB^	Nd
US 50 kHz + AA 1000 mg/L	5.0 ± 0.2 ^aBCD^	5.0 ± 0.8 ^aB^	3.1 ± 0.3 ^aD^	3.9 ± 0.5 ^aB^	2.1 ± 0.2 ^BC^	Nd
US 50 kHz + AA 2000 mg/L	4.3 ± 0.4 ^aD^	5.0 ± 0.6 ^aB^	3.3 ± 0.2 ^aD^	3.7 ± 0.3 ^aB^	2.2 ± 0.2 ^BC^	Nd
US 50 kHz + PA 20 mg/L	4.2 ± 0.1 ^aD^	4.6 ± 0.3 ^aB^	3.0 ± 0.2 ^aD^	3.4 ± 0.2 ^aB^	1.2 ± 0.3 ^C^	Nd

Means on the same line followed for the same lowercase letter do not differentiate between each other (*p* > 0.05), for each microorganism, in the Tukey test after three replications. Means in the same column followed for the same capital letter do not differentiate between each other (*p* > 0.05) in the Tukey test after three replications. SDC: sodium dichloroisocyanurate; AA: acetic acid; PAA: peracetic acid; US: ultrasound; Nd: not detected in the smallest plated dilution (10^−1^).

**Table 2:** Average and standard deviation of pH, total titratable acidity, and total soluble solids in kale samples after sanitization and over storage at 7 °C.

Treatment	pH		TTA (mg Citric Acid/100 mg)		TSS (°Brix)	
	Day 1	Day 6	Day 1	Day 6	Day 1	Day 6
Non-sanitizer	6.7 ± 0.3 ^aAB^	6.8 ± 0.1 ^aA^	0.1 ± 0.01 ^aC^	0.08 ± 0.01 ^aB^	7.5 ± 0.5 ^aA^	6.1 ± 0.3 ^aA^
SDC 200 mg/L	6.8 ± 0.2 ^aA^	6.8 ± 0.2 ^aA^	0.1 ± 0.01 ^aC^	0.10 ± 0.01 ^aB^	6.2 ± 0.4 ^aABC^	6.2 ± 0.3 ^aA^
AA 1000 mg/L	5.3 ± 0.1^aBC^	5.6 ± 0.4 ^aAB^	0.4± 0.01 ^aABC^	0.26 ± 0.02 ^aAB^	4.9 ± 0.3 ^aBC^	5.9 ± 0.3 ^aA^
AA 2000 mg/L	5.0 ± 0.1 ^aC^	4.7 ± 0.1 ^bB^	0.5 ± 0.08 ^aA^	0.49 ± 0.11 ^aA^	5.7 ± 0.3 ^aBC^	5.8 ± 0.1 ^aA^
PAA 20 mg/L	6.8 ± 0.2 ^aAB^	6.9 ± 0.2 ^aA^	0.2 ± 0.02 ^aBC^	0.10 ± 0.02 ^aB^	6.1 ± 0.1 ^aABC^	6.1 ± 0.5 ^aA^
US 50 kHz	6.8 ± 0.2 ^aAB^	7.0 ± 0.2 ^aA^	0.1 ± 0.01 ^aC^	0.14 ± 0.03 ^aB^	6.9 ± 0.4 ^aAB^	5.8 ± 0.4 ^aA^
US 50 kHz + AA 1000 mg/L	5.8 ± 0.7 ^aABC^	6.1 ± 0.8 ^aAB^	0.3 ± 0.12 ^aABC^	0.28 ± 0.10 ^aAB^	5.4 ± 0.1 ^aBC^	5.8 ± 0.2 ^aA^
US 50 kHz + AA 2000 mg/L	5.1 ± 0.4 ^aC^	4.9 ± 0.1 ^aB^	0.4 ± 0.06 ^aAB^	0.53 ± 0.07 ^aA^	5.9 ± 0.2 ^aABC^	6.4 ± 0.4 ^aA^
US 50 kHz + PAA 20 mg/L	6.8 ± 0.2 ^aAB^	6.9 ± 0.1 ^aA^	0.1 ± 0.01 ^aC^	0.13 ± 0.03 ^aB^	5.6 ± 0.4 ^aBC^	5.7 ± 0.6 ^aA^

Means on the same line followed for the same lowercase letter do not differentiate between each other (*p* > 0.05) in the Tukey test after three replications. Means in the same column followed for the same capital letter do not differentiate between each other (*p* > 0.05) in the Tukey test after three replications. TTA: total titratable acidity; SDC: sodium dichloroisocyanurate; AA: acetic acid; PAA: peracetic acid; US: ultrasound.

**Table 3:** Average and standard deviation of vitamin C (mg of ascorbic acid/100 g), phenolic compounds (mg gallic acid equivalent/100 g), and antioxidant activity (%) in kale samples after sanitization and storage at 7 °C.

Treatment	Vitamin C		Total Phenolic Compounds		Antioxidant Activity	
	Day 1	Day 6	Day 1	Day 6	Day 1	Day 6
No sanitizer	56.0 ± 4.0 ^aA^	50.3 ± 6.9 ^aA^	21.9 ± 2.8 ^aA^	25.1 ± 3.2 ^aA^	92.6 ± 6.4 ^aA^	56.5 ± 11.9 ^bA^
SD 200 mg/L	52.9 ± 2.9 ^aA^	45.2 ± 2.0 ^aA^	21.4 ± 1.2 ^aA^	23.9 ± 4.6 ^aA^	87.9 ± 5.2 ^aA^	41.9 ± 7.7 ^bA^
AA 1000 mg/L	51.5 ± 1.9 ^aA^	41.4 ± 8.2 ^aA^	19.4 ± 2.5 ^aA^	24.1 ± 5.1 ^aA^	84.6 ± 13.6 ^aA^	29.3 ± 6.1 ^bA^
AA 2000 mg/L	52.1 ± 4.3 ^aA^	47.3 ± 4.7 ^aA^	24.3 ± 0.7 ^aA^	23.0 ± 5.5 ^aA^	76.0 ± 7.5 ^aA^	33.1 ± 3.3 ^bA^
PAA 20 mg/L	48.5 ± 9.1 ^aA^	48.2 ± 10.8 ^aA^	22.5 ± 3.5 ^aA^	20.1 ± 2.4 ^aA^	89.9 ± 11.1 ^aA^	40.1 ± 7.0 ^bA^
US 50 kHz	48.3 ± 2.9 ^aA^	46.5 ± 8.6 ^aA^	20.2 ± 5.3 ^aA^	23.0 ± 5.3 ^aA^	96.9 ± 2.4 ^aA^	40.9 ± 8.0 ^bA^
US 50 kHz + AA 1000 mg/L	51.9 ± 3.5 ^aA^	52.9 ± 6.6 ^aA^	23.7 ± 1.9 ^aA^	23.8 ± 5.1 ^aA^	96.3 ± 3.3 ^aA^	35.0 ± 6.5 ^bA^
US 50 kHz + AA 2000 mg/L	56.3 ± 6.3 ^aA^	53.6 ± 7.3 ^aA^	21.1 ± 1.1 ^aA^	25.7 ± 2.5 ^aA^	96.0 ± 3.9 ^aA^	33.8 ± 5.5 ^bA^
US 50 kHz + PAA 20 mg/L	48.4 ± 10.5 ^aA^	46.0 ± 11.1 ^aA^	19.2 ± 1.4 ^aA^	26.2 ± 6.9 ^aA^	96.6 ± 2.1 ^aA^	41.8 ± 5.6 ^bA^

Means on the same line followed for the same lowercase letter do not differentiate between each other (*p* > 0.05) in the Tukey test after three replications. Means in the same column followed for the same capital letter do not differentiate between each other (*p* > 0.05) in the Tukey test after three replications. SDC: sodium dichloroisocyanurate; AA: acetic acid; PAA: peracetic acid; US: ultrasound.

## Data Availability

The data that support the findings of this study are available on request from the corresponding author.

## Sample Availability

Samples of the compounds are not available from the authors.
